# Evaluation of RFID Tags to Permanently Mark Trees in Natural Populations

**DOI:** 10.3389/fpls.2016.01342

**Published:** 2016-08-31

**Authors:** Tobias Marczewski, Yongpeng Ma, Weibang Sun

**Affiliations:** Key Laboratory for Plant Diversity and Biogeography of East Asia, Kunming Institute of Botany, Chinese Academy of SciencesKunming, China

**Keywords:** long-range detection, marking, RFID, tagging, tree, tree population

## Abstract

Long-term ecological and genetic studies in natural populations of tree species require marking techniques so that individuals can be re-visited over time, even in difficult terrain. Both GPS coordinates and physical labels have disadvantages that can make re-finding trees difficult. We tested passive and semi-active radio frequency identification (RFID) tags and readers as a means to relocate individual trees. Passive RFID tags do not provide a good solution because of low transmission power of hand-held readers and strong directionality. Semi-active RFID tags provide detection over longer distances, but also suffer from strong directionality. Active RFID tags promise an improvement over semi-passive tags, and could be evaluated in a future study. We conclude that RFID technology has the potential to improve the ability of researchers to locate individual trees repeatedly under natural conditions, and can be used in conjunction with other marking techniques such as physical tags and GPS coordinates.

## Introduction

Studies concerned with tree species growing in natural populations are carried out in several fields of biology, including different branches of ecology as well as genetics. Many of these studies require repeated data collection involving the same individuals, which makes it essential that they can be located repeatedly over the course of the investigation or experiment. Depending on the nature of the study it will be desirable to sub-sample a forest community, which is generally done by establishing plots in selected areas and marking each individual with a persistent, unique, label (Condit, 1998). In such a setting the grid structure of the plot can provide the means to relocate individual trees, especially when an adequate labeling scheme is used, that incorporates the layout of the grid (Condit, 1998). Plots covering a large area are, however, very time and resource consuming to set up, and might not meet the needs of a particular question. If, for example only a single species is of interest, it might be important to include many individuals of this species, rather covering a large area than restricting the sampling to a plot, especially if the population density is low.

Another case is if only certain individuals are of interest, based for example on morphological traits or genetic composition. A timely example would be genetic studies that are increasingly feasible in non-model organisms due to rapidly advancing sequencing technologies. Genetic mapping and gene-phenotype association studies require adequate mapping populations comprising crosses of divergent genetic backgrounds. For plants with short generation times, such populations can be produced artificially under controlled greenhouse conditions with relative ease. Furthermore, due to short generation times, later-generation crosses such as F2s, F3s, and backcrosses, can also be obtained in reasonable time. However, this is not the case for woody plants with long generation times, where individuals can take several years before entering the reproductive phase. To overcome this issue, the use of naturally occurring hybrid populations has been suggested (Lexer et al., 2004; Buerkle and Lexer, 2008). Such populations can already comprise several generations of later-generation crosses, and thus can potentially provide the means for mapping in plants with longer generation times. Furthermore, individual trees can persist over several decades, and by means of cross-pollinating individuals with known genetic background, including hybrids, times to obtain adequate artificial mapping populations could be shortened considerably.

Crucial for all studies that either want to obtain data repeatedly, or use plants for follow-up experiments, is the unequivocal location and identification of the individuals from which information was obtained in the initial sampling. Hence the trees must be marked in a manner that allows them to be re-visited over the course of the experiment.

The method most widely used in botany to mark trees is to employ physical labels, for example numbered metal plates. This alone, however, does not provide information about the location of the plant. Therefore, hand-drawn maps are additionally employed, which are feasible mostly when working in a plot setting, or GPS coordinates are used as a proxy for the location. While a location obtained from a GPS reading is certainly accurate enough to mark a larger area, the accuracy of the reading can be greatly influenced by external factors, such as weather, terrain and canopy cover. Particularly for tree species growing in dense forest, GPS coordinates often have confidence intervals that are large (up to 10 m, occasionally the confidence circle will not even overlap with the last taken coordinates of the individual; T. Marczewski pers. obs.), and cannot be used as a secure method to locate trees after initial sampling.

Hence, to better locate individuals, trees should be additionally marked in a way that bridges the distance from a GPS coordinate to the actual individual. Paint or brightly colored duct-tape can provide visual clues that can increase re-finding considerably (T. Marczewski, pers. obs.), but can also fail under certain conditions. Visual clues might be eroded away over time, or in certain light conditions, for example dappled light under canopy cover, might not be as visible as desired. In case of a lost visible clue (or plant) the inaccuracy of the position obtained by GPS coordinates can lead to a verification problem, as the loss of the marker will be unknown during the attempt to re-locate the tree. Hence, if a previously marked individual has not been found after a certain search time, the possibility of loss has to be considered, which might lead to marked trees being overlooked if the individual was not found because of inaccurate initial coordinates. Therefore, a marker that would be detectable over longer distances with other means, not relying on visual perception alone, could enhance location of individuals.

For over two decades, Radio Frequency Identification (RFID) has been used by zoologists to tag animals and allow identification of individuals (Bonter and Bridge, 2011). RFID tags are a microchip with an attached antenna, and together with an appropriate reader, data can be read from the chip. In passive RFID tags no battery is present, and the energy required to power the chip is provided by the radio signal energy emitted by the reader. Semi-active tags contain a battery that powers tag transmission in response to the signal of a reader, resulting in greater transmission distances. Active RFID tags transmit at set intervals even when not responding to the signal of a reader, and have the greatest transmission distances. In all RFID tags detecting the transmission signal depends on the reader, the distance between the reader and the tag, and the size of the antenna of the tag. Additionally, in passive and semi-passive RFID tags the size of the tag antenna determines how much of the power emitted by the reader will be available (through induction) to the microchip.

Radio frequency identification tags used to mark animals are usually as small as possible, so that when the tags are injected under the skin or otherwise attached to the animal, they have as little impact on the individual as possible. The small size of these tags, and thus the antennae, means that only at short distances (<0.3 m) will the energy emitted by the reader be sufficient to power the microchip and enable it to broadcast back. In some cases where locating individuals is not of prime concern, implanted short-distance RFID tags have also been employed in woody plant species, mostly to avoid loss of tags (Bowman, 2010).

Because passive RFID tags do not contain a battery, they have the advantage that they can theoretically function for several decades, and the location (direction from the reader) of a tag that broadcasts can be determined with a reader having the appropriate software installed. Therefore, RFID tags could potentially provide a means to home in on a tagged individual, after the rough location has been determined with a GPS device. As mentioned before, the minute tags (in the mm range) used in zoological applications have a very limited detection range, and to bridge inaccurate GPS readings efficiently it would be desirable to be able to detect a given tag from at least 10 m distance. For the marking of trees, the size of the tags is, however, not as restricted as in zoological applications, as possible effects on the marked individual do not have to be considered.

In this study, we wanted to assess if passive- or semi-passive RFID tags that have comparably large nominal read-ranges have large enough transmission distances to be useful for re-locating trees under natural field conditions, using hand-held readers. We tested (1) realized detection distances under optimal conditions (flat area) from different angles, and (2) performance in the field in dense forest in (a) even terrain and (b) hilly terrain.

## Materials and Methods

We tested three different types of robust hand-held readers, and three different types of RFID tags which seemed to meet the requirements according to the manufacturers specifications. The readers, we tested were a commercial model used for industrial applications (Motorola MC9190-Z; US frequency antenna: 902-928 MHz; 3 Watt ERP), an antenna that can be attached to a smart phone (Grokker; US frequency; ERP not specified), and a custom made device by a small company (Trolley Scan Handheld; uses customized tags). The commercial and smart phone readers were tested with three types of RFID tags (**Supplementary Figure S1**): passive medium-size (Omni-ID Dura 1500; size: 14 cm × 6.6 cm; fixed reader range ≤ 15 m, hand-held ≤ 7.5 m), passive large-size (Omni-ID Dura 3000; size: 21 cm × 11 cm; fixed reader range ≤ 35 m, hand-held ≤ 20 m), and a semi-passive medium-size (Omni-ID Power 50; size: 13.9 cm × 6.6 cm; fixed reader range ≤ 50 m, hand-held not stated). The semi-passive tag actually contains a battery to provide extra power if energy from a broadcasting reader is received, and has an estimated 5 years lifespan.

All work was carried out in Baili Scenic Reserve, Guizhou, China in March 2016. Two types of tests were conducted. Firstly, a tag was fixed to a tree (stem diameter ∼25 cm) using metal wire (**Supplementary Figure S2**), and then the minimum distance needed to obtain a signal with the reader (Geiger-counter like noise) was measured in 45° steps around the tree. The distance was tested by going toward the tag, so that the tag would not be powered beforehand, which might have resulted in a larger distance. For the second test only the reader-tag combination that achieved the largest overall detection distance was used; ten trees were tagged and inaccurate GPS coordinates were taken by one team (coordinates were taken from a position obtained by walking 2–4 m in a random direction from the tree in question), while a second team tried to locate the trees using a GPS and reader. The tags were hidden repeatedly in forest with dense undergrowth, first in more or less level terrain, and second in hilly terrain on various slopes.

## Results

The reader from the small company (Trolley Scan) turned out to be unsuitable because the tags provided with the reader were detectable only from under 0.5 m. It was not possible to use this reader in conjunction with tags from a different company, which made it impossible to employ tags that might have been better suited. This reader was therefore not tested further. The antenna-smart-phone combination had apparently very little output power, and the tags only received enough energy to broadcast back from very short distances (about 1 m, **Table 1**). Only the large-passive tag was detected from a greater distance (up to 7 m), but only when directly facing the tag. The commercial reader enabled detection from considerably larger distances, again, the large-passive tag was recognized from furthest away (∼13 m, **Table 1**).

**Table 1:** Detection distances (in m) for tested reader-tag combinations when affixing the tag to a tree.

Reader	Mobile-phone-connected (Grokker)			Commercial reader (Motorola)		
		
Tag	Large	Medium	Semi-passive	Large	Medium	Semi-passive
**Orientation**						
0°	7.06 (±0.63)	1.38 (±0.16)	1.08 (±0.13)	13.04 (±2.16)	4.56 (±1.19)	8.78 (±0.15)
45°	1.99 (±0.69)	1.12 (±0.13)	1.18 (±0.18)	8.64 (±1.29)	4.66 (±0.88)	6.06 (±0.22)
90°	0.48 (±0.22)	0.52 (±0.14)	0.68 (±0.42)	5.18 (±0.91)	1.90 (±0.44)	5.34 (±0.60)
135°	0.36 (±0.26)	0.41 (±0.11)	0.33 (±0.10)	1.46 (±0.17)	2.24 (±0.86)	3.76 (±0.11)
180°	0.20 (±0.12)	0.47 (±0.13)	0.56 (±0.40)	0.66 (±0.18)	1.72 (±0.08)	4.34 (±0.57)
225°	0.29 (±0.11)	0.51 (±0.19)	0.58 (±0.45)	1.52 (±0.26)	1.74 (±0.37)	3.14 (±0.31)
270°	0.74 (±0.20)	0.96 (±0.18)	0.68 (±0.33)	1.08 (±0.31)	2.48 (±0.34)	3.58 (±0.50)
315°	2.54 (±0.43)	1.01 (±0.11)	0.70 (±0.12)	4.72 (±1.24)	3.98 (±0.18)	4.94 (±1.23)


*Mean (± Standard deviation, *N* = 5), measured in 45° angle steps, where 0° means facing the tag.*

**FIGURE 1:**
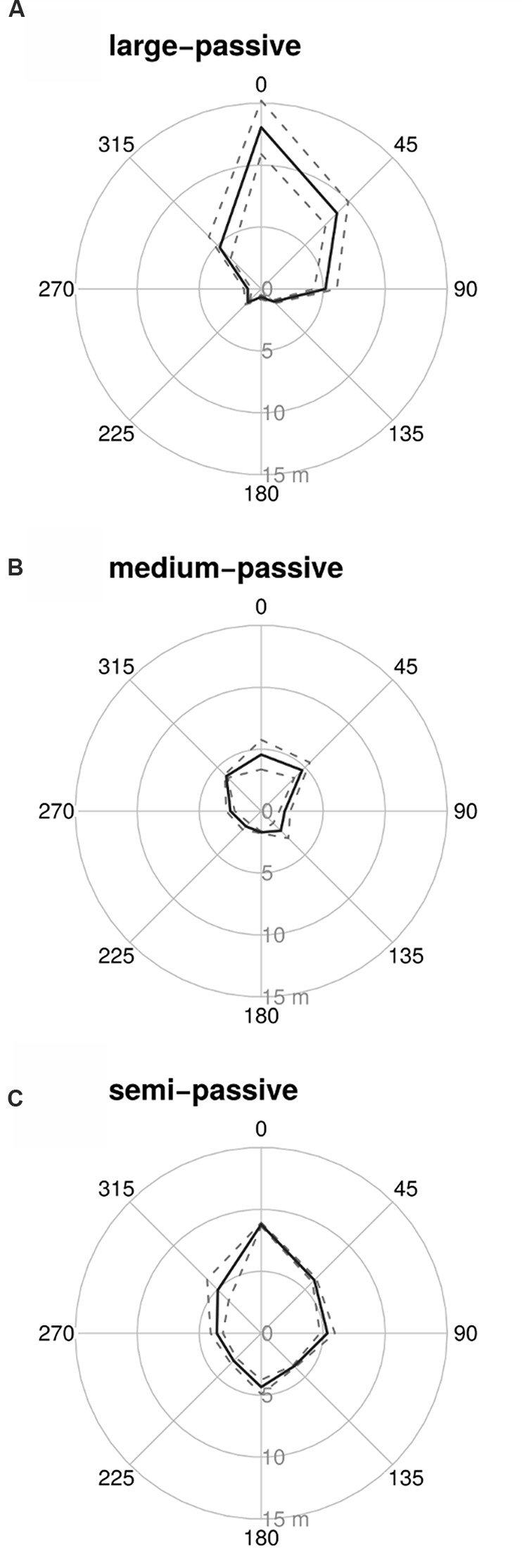
**Detection distances for the three tested tag types [**(A)** – large passive tag, **(B)** – medium passive tag, and **(C)** – semi-passive tag], using the commercial reader (Motorola).** To obtain these measurements the tags were affixed to a tree with the tag facing in direction 0°. For every tag, five measurements were taken for each angle. Shown are the mean detection distance (solid line) and one standard deviation (dashed line).

Although the large-passive tag provided the longest detection range when the reader was facing the tag (**Figure 1A** at 0°), all tags tested suffered from strong directionality effects with rapid drops in detection range when not facing the tag. Tags were detectable only from very short distances (<1 m) when the reader was positioned behind the tag (**Table 1**; **Figure 1** at 180°). The semi-active tag was less affected by directionality, but the detection distance still dropped to less than half the maximum distance when not facing the tag (**Table 1**; **Figure 1C**). None of the tags could provide a minimum detection range of >10 m from any direction. A semi-passive version of the large tag was available, but was not tested, and this might have given a read-range of 10 m. However, the dimensions of the large tag make it only suitable for marking larger trees (e.g., >50 cm diameter).

The most suitable tag (least affected by directionality effects) seemed the semi-passive tag, in combination with the commercial reader. When, we tested this combination in the field, using GPS and reader together, the signal helped significantly with the location of marked trees on level ground. We allowed a search time of 10 min per tag, but most were located within 2 min, starting from the GPS coordinates. However, even then, two of 40 tags were not found. After switching to hilly terrain the short detection distance came into play even more severely, and in the first hiding attempt (10 tags), only five were found by the searching team, with two of the non-located tags being lost for good. The assessment of suitability in hilly terrain was after this attempt not pursued further, as the performance of the tags was obviously insufficient. Problems with re-locating the tags were partly attributable to the GPS coordinates being too inaccurate under the given terrain conditions, sometimes having confidence deviations of >15 m, which the short detection distances could not bridge.

## Conclusion

The combination of reader and tags by Trolley Scan failed before tests in the field were conducted. The biggest problem was that only custom tags could be used with this reader, therefore we suggest avoiding customized readers that do not use standard frequencies, as the restricted choice of tags will not allow the use of tags that best fit the intended purpose.

Passive RFID tags seemed a good choice for marking as they are not restricted by battery life and are considerably cheaper in comparison to active tags. However, due to achieved distances and directionality problems they are not suitable. This is unlikely to improve in the future, as the only means to provide longer detection distances are larger tags not useful for smaller trees, or readers that provide higher output power, which is restricted for field-suitable hand-held readers.

Despite the unsuitability of passive tags for long-distance detection, we still believe that RFID technology has potential to mark trees in the wild, and thus could help making natural populations more accessible for experiments. In setups where the locations of trees are relatively well known, for example for well designed grids in plot experiments, but persistence of labels is of major concern, implantable RFID tags (Bowman, 2010) might be more suitable and cost effective (<$1 per tag) than external tags. If researchers face the same problems of re-location as outlined in this study, it would be worthwhile to investigate the performance of active tags, which we did not assess due to financial and time restrictions. The semi-passive (battery aided) tag showed significantly better behavior than the passive variants, and should be easily surpassed by active tags. The problem of battery-life in active tags could also be lessened for the intended application (marking trees), as the broadcasting frequency of many tags can be specified before ordering. Under default settings tags will broadcast every 3–5 s, resulting in a battery-life of about 3 years, if this would be set for example to about 30 s, the life span should at least double, with minimal effects to the intended use.

The use of RFID tags will necessarily increase the cost of marking trees, especially if active tags are employed (∼$10 per tag, <$1000 for one reader), but this expense might be worthwhile when relocating trees allows the collection of critical or otherwise expensive data. For example in comparison to the current costs to obtain high-density coverage next-generation-sequencing data for a single individual (∼$100), the cost of RFID equipment will be relatively low.

We would advise fellow researchers facing the problem of marking dispersed individuals in tree populations, be it for ecological, genetic or other studies, to employ active tag-reader combinations, either in the 2.45 GHz or the 433 MHz band, to supplement established marking techniques.

## Author Contributions

TM and YM planned and designed the research, and performed experiments; TM wrote the manuscript; WS provided funding.

## Conflict of Interest Statement

The authors declare that the research was conducted in the absence of any commercial or financial relationships that could be construed as a potential conflict of interest.
