# Supplementary material for: Evaluation of RFID Tags to Permanently Mark Trees in Natural Populations
Source: Front Plant Sci. 2016 Aug 31;7:1342. doi: 10.3389/fpls.2016.01342 (PMC5006006; doi:10.3389/fpls.2016.01342)
Supplement: Supplementary file 1 [file Image_1.PDF]

Supplementary Material

Evaluation of RFID tags to permanently mark trees in natural populations

Tobias Marczewski[1*†], Yongpeng Ma[1†] and Weibang Sun[1*]

[1]Key Laboratory for Plant Diversity and Biogeography of East Asia, Kunming Institute of Botany, Chinese Academy of Sciences, Kunming, Yunnan, China

*** Correspondence:**
Tobias Marczewski
Email: tmarczewski@mail.kib.ac.cn
Weibang Sun
Email: wbsun@mail.kib.ac.cn

[†] These authors contributed equally to this work.

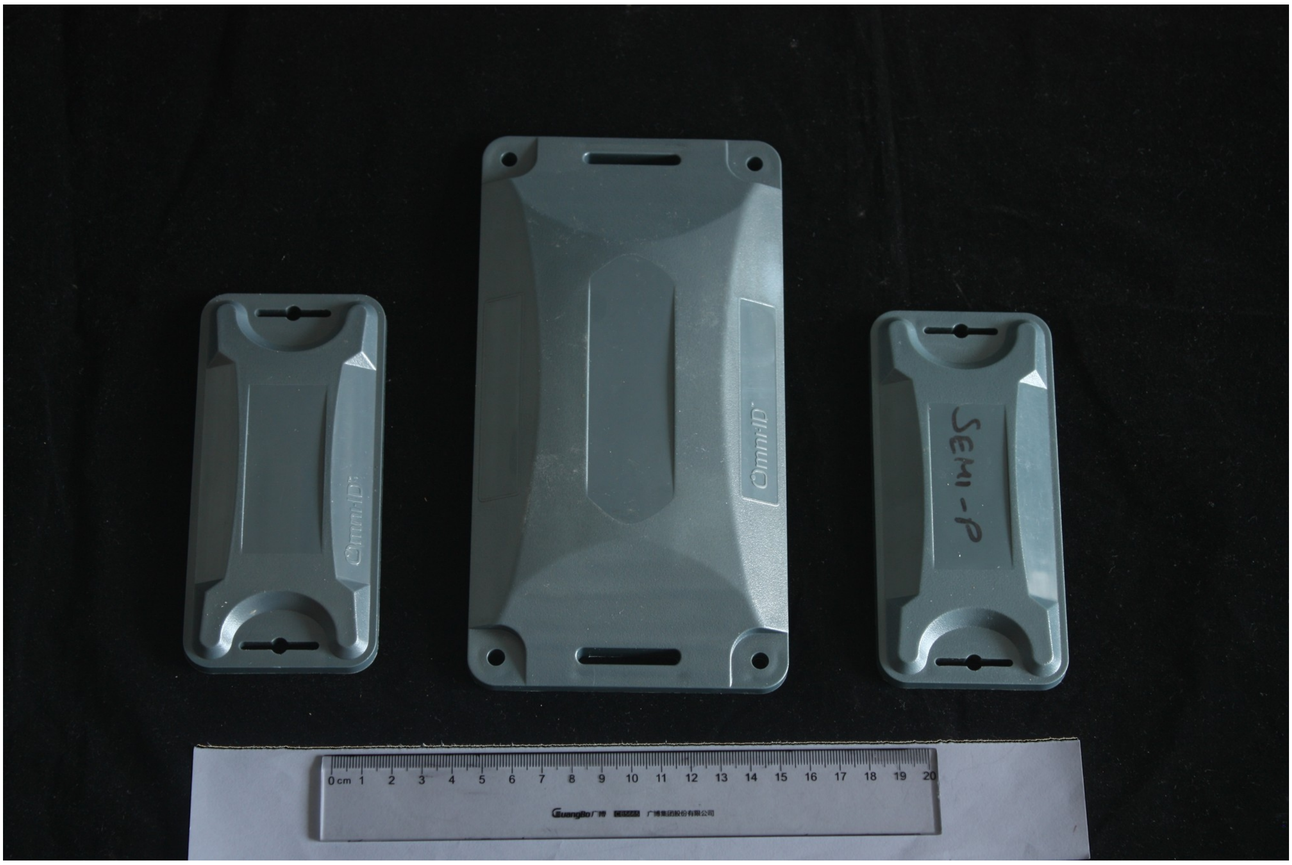

Supp Fig. 1 RFID tags tested in the study. From left to right: medium passive tag, large passive tag, semi-passive tag.

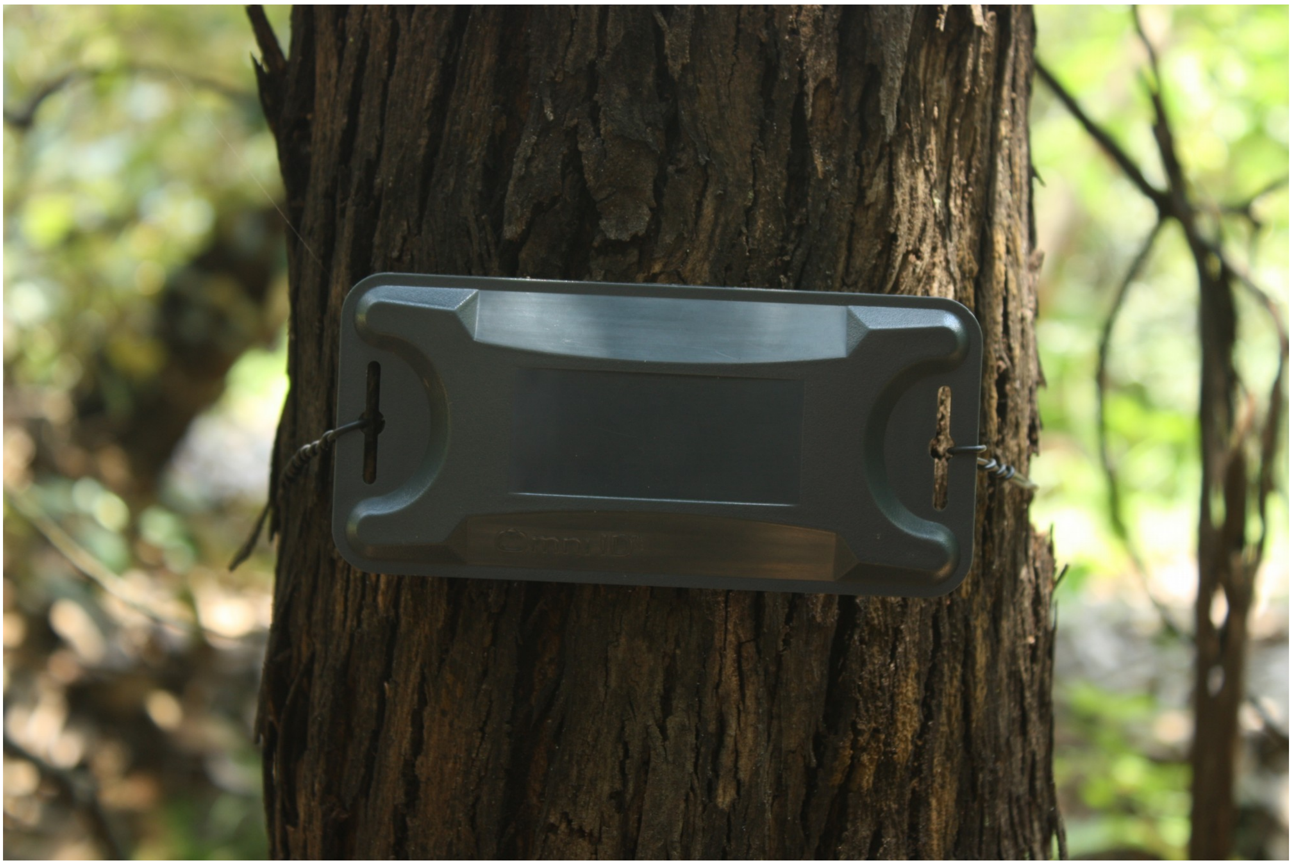

Supp Fig. 2 Medium passive tag fastened to tree with metal wire.
